# Prominent Effects of Berbamine Hydrochloride on Alzheimer’s Disease Model Mice

**DOI:** 10.3389/fphar.2022.939039

**Published:** 2022-06-30

**Authors:** Chang-lan Chen, Hai-li Wang, Feng Qian, Mei-hua Liu, Xiao-xuan Guo, Jing Lv, Jia-hui Huang, Nai-zhi Zhang, Zheng Xiang

**Affiliations:** ^1^ School of Light Industry, Liaoning University, Shenyang, China; ^2^ School of Pharmaceutical Science, Liaoning University, Shenyang, China

**Keywords:** Alzheimer’s disease, berbamine hydrochloride, Morris water maze, β-amyloid, neurofibrillary tangle, calpain, selenoprotein K

## Abstract

Very few anti-Alzheimer’s disease (AD) drugs are clinically available at present due to the complex mechanism of Alzheimer’s disease. For the purpose of discovering potential anti-AD drugs in bisbenzylisoquinoline alkaloids, the anti-AD function and the mechanism of the function of berbamine hydrochloride (BBMH) were studied. Three kinds of AD model mice, double transgenic APP/PS1 AD mice, Gal-Alu AD mice induced by the intraperitoneal injection of d-galactose combined with the intragastric administration of aluminum trichloride, and Alu AD-like mice induced by stereotactic brain injection of aluminum trichloride, were administered with BBMH for 40 days at a dosage of 280 mg/kg/d. The effects of BBMH on the learning and memory behavior of the AD mice were studied through the Morris water maze experiment, and the influences of BBMH on the pathological features of AD, including the deposition of Aβ, the lesions of pyramidal cells (neurons), and the formation of neurofibrillary tangles, were studied by the immunohistochemical staining, hematoxylin-eosin staining, and silver staining of the brain tissues of the mice. The water maze experiment showed that BBMH could significantly improve the learning and memory abilities of three kinds of treated mice. Immunohistochemical staining showed that BBMH could significantly reduce the deposition of Aβ in the brain tissues of treated mice. Hematoxylin-eosin staining showed that BBMH could significantly alleviate the lesions of pyramidal cells in the hippocampal tissue of the mice. Silver staining showed that BBMH could significantly reduce the formation of neurofibrillary tangles in the hippocampal tissue of the mice. These results indicated that BBMH has significant anti-AD effects and the potential as an anti-AD drug. Western blot analysis of the brain tissue of the mice showed that the expression level of calpain, a Ca^2+^-dependent proteolytic enzyme, was significantly inhibited and the expression level of SelK, a selenoprotein mainly expressed in immune cells, was significantly increased. It is speculated that the anti-AD effect of BBMH is related to the improvement of the phagocytosis of microglial cells in brain tissues and macrophages migrated into the brain as well as the regulation of calcium homeostasis and calcium-dependent proteases in the brain tissues of the mice.

## 1 Introduction

Alzheimer’s disease (AD) is a disease with high incidence in the elderly, which seriously endangers the life quality of people (Lane et al., 2018). Unfortunately, due to its complex etiology, currently there are limited anti-AD drugs that can be used clinically (Ju et al., 2021). Many hypotheses about the pathogenesis of the disease have been proposed by researchers, including the amyloid cascade hypothesis (Hardy and Higgins, 2019), presenilin hypothesis (Shen and Kelleher, 2007), APP matrix hypothesis (Hunter and Brayne, 2018), tau hyperphosphorylation hypothesis (Iqbal et al., 2016), neuroinflammation hypothesis (Heppner et al., 2015), oxidative stress hypothesis (Cheignon et al., 2018), mitochondrial dysfunction hypothesis (Pei and Wallace, 2018), ER stress hypothesis (Hetz and Saxena, 2017), glucose regulation disorder hypothesis (Neth and Craft, 2017), and cholesterol and metal metabolism hypothesis (Wong et al., 2014).

The discovery of potential drugs derived from natural products is an important way to develop new drugs (Chen C. et al., 2021; Wang et al., 2021). Bisbenzylisoquinoline alkaloids such as liensinine, isoliensinine, puerarin, tetrandrine, and berbamine have neuroprotective and anti-inflammatory effects mainly as calcium antagonists (Sharma et al., 2017; Chan et al., 2021; Kim et al., 2021; Guo et al., 2022). Previous studies indicated that liensinine and isoliensinine exhibited certain anti-AD effects (Meng et al., 2019). Berbamine (BBM), one of the bio-active alkaloids, is often present in the form of berbamine hydrochloride (BBMH). BBMH has been clinically used to promote leukocyte hyperplasia, anti-tumor, and protect the immune system and cardiovascular system (Xiping et al., 2016; Jia et al., 2017; Zhang et al., 2012).

For the purpose of studying the neuroprotective effects of BBMH on AD, three kinds of AD model mice including double transgenic APP/PS1 AD mice, Gal-Alu AD mice induced by the intraperitoneal injection of d-galactose combined with the intragastric administration of aluminum trichloride, and Alu AD mice induced by stereotactic brain injection of aluminum trichloride were prepared first. Then, they were administered with BBMH for 40 days at a dosage of 280 mg/kg/d by gavage according to the previously reported (Wang et al., 2006) and the results of the pre-experiment. Subsequently, the effect of BBMH on the learning and memory ability of AD mice was investigated through the Morris water maze experiment, and the influence of BBMH on the state of pyramidal cells and the formation of Aβ deposition was studied by hematoxylin-eosin staining and immunohistochemical staining of mouse brain hippocampal tissue. The influence of BBMH on the formation of neurofibrillary tangles in mouse hippocampal tissue was investigated by glycine silver staining. Finally, the effect of BBMH on the expression of Ca^2+^-dependent proteolytic enzyme, calpain, and its target protein, selenoprotein SelK, was tested by the method of Western blot.

## 2 Materials and Methods

### 2.1 Drugs and Reagents

BBMH was purchased from Chengdu Delico Biotechnology Co., LTD. Streptavidin-HRP kit (DAB), SP rabbit HRP kit (DAB), citrate buffer (100×), and BCA protein assay kit were purchased from Beijing Kang Wei Century Biotechnology Co., LTD. Sodium pentobarbital, edible melanin, and physiological saline were purchased from Shenyang Laibo Science and Trade Co., LTD. d-Galactose was purchased from Beijing Dingguo Changsheng Biotechnology Co., LTD. Anhydrous ethanol, n-butanol, and xylene were purchased from Kelon Chemical Reagent Factory in Chengdu. Hematoxylin dye and neutral resin were purchased from Beijing Dingguo Changsheng Biotechnology Co., LTD. Anti-fluorescence quenching sealing tablets and aluminum chloride were purchased from Shanghai Maclin Biochemical Technology Co., LTD. Bovine serum albumin (BSA) was purchased from Beijing Solebo Technology Co., LTD. Silver glycine dye set, tissue fixative fluid, and HE dye were purchased from Wuhan Xavier Biotechnology Co., LTD. First antibody of rabbit anti-beta-amyloid 1–40 (CT) antibody and rabbit anti-β-actin were purchased from Beijing Biosynthesis Biotechnology Co., LTD. First antibody of rabbit calpain two polyclonal antibody and rabbit SELK polyclonal antibody were purchased from Proteintech Inc. Secondary antibodies of HRP-conjugated goat anti-rabbit antibody was purchased from Beyotime Co., LTD. The ECL reagents were purchased from Tanon (Shanghai, China).

### 2.2 Animals and Treatments

#### 2.2.1 Preparation of APP/PS1 Transgenic Mice and BBMH Administration of the Mice

The parental APP/PS1 mice were donated by PLA Northern Theater Command Hospital. The APP/PS1 offspring mice were generated by the crossing of parental APP/PS1 mice. The double transgenic progeny of APP/PS1 mice were identified by a mouse tail gene identification kit (Lin et al., 2018). Twelve six-month-old APP/PS1 male mice were randomly divided into two groups, the administration group and the model group. The mice in the administration group were given 280 mg/kg/d (suspended in physiological saline solution at 1:4 w/w) BBMH for 40 days by gavage, and the model AD group and normal control mice (6-month-old wild-type mice) were given the same amount of physiological saline solution for 40 days.

#### 2.2.2 Preparation of Gal-Alu AD Model Mice and BBMH Administration of the Mice

The male mice were purchased from Liaoning Changsheng Biotechnology Co., LTD (Kunming male SPF mice, Animal License: SCXK Liao, 2010–0001) at the age of 6–7 weeks (weight 20–22 g). For the purpose of preparing AD model mice, they were given 20 mg/kg/d aluminum chloride solution by gavage and 120 mg/kg/d d-galactose solution (dissolved in normal saline solution) intraperitoneally injected continuously for 40 days according to the method (Xing et al., 2018) with minor modification. Then, 16 modeled AD mice were randomly divided into two groups including the BBMH administration group and the Gal-Alu model AD group. The former was given BBMH solution (dissolved in normal saline) at the dosage of 280 mg/kg/d by gavage for 40 days, and the latter was given the same amount of normal saline for 40 days, respectively.

#### 2.2.3 Preparation of Alu AD Mice and BBMH Administration of the Mice

After being anesthetized by the intraperitoneal injection of 4% sodium pentobarbital at a dosage of 0.025 ml/10 g, the male mice were fixed on the brain stereotaxic instrument and 3 μl of 2.5% aluminum trichloride was injected into the brain hippocampal tissues of the mice using a microsyringe (Zhang and Yu, 2002). The sham operation group was injected with an equal volume of saline. After being fed for another 2 months, they were randomly divided into two groups including the BBMH administration group and the Alu model AD group. The former was given BBMH at the dosage of 280 mg/kg/d by gavage for 40 days, while the latter was given the same amount of normal saline for 40 days, respectively.

### 2.3 Measurement of the Learning and Memory Abilities of Mice by the Morris Water Maze Test

The Morris water maze test was conducted as described in the previous work (Tang et al., 2020) with minor modifications. There are I, II, III, and VI quadrants in the Morris water maze (Shanghai XinRuan Information Technology Co., Ltd., Shanghai, China) for mice. The platform was fixed in the center of the third quadrant (target quadrant) and submerged approximately 1 cm beneath the water surface. The training trial was repeated for four consecutive days. For each trial, the mice were gently released from the first to fourth quadrants; sequentially, the mice were put gently into the water (temperature: 22 ± 1°C) facing the wall of the water tank and avoiding making noise and other disturbances to the mice. The trial was terminated if the mouse failed to climb onto the platform within 90 s. Also, the time to reach the platform (escape latency) and path length of the mice were automatically recorded using a video recording system. If the mouse did not find the platform within 90 s, it was guided to the platform and stayed there for 20 s, and the escape latency was recorded as 90 s.

The probe trial was administered 24 h after the last training session. In the probe trial, the platform was removed from the pool, and the mice were gently placed in the opposite quadrant of the third quadrant (the target quadrant), facing the wall of the water tank. Then, the time of mice spent swimming in the third quadrant housing the platform (target quadrant) within 90 s and the number of crossing the previous platform location in the target quadrant were measured using an automated analysis system.

### 2.4 Preparation of the Paraffin Section

After being anesthetized by the intraperitoneal injection of 4% sodium pentobarbital, the mice were then killed by removal of the neck, and the whole brain tissues of five mice for every treatment were taken out and put into 100 ml tissue fixation solution for more than 24 h. The fixed brain tissues of mice were soaked in 70, 85, 95, and 100% ethanol for several hours successively for dehydration and then soaked in a mixture of xylene and ethanol (V/V, 50/50) for 10 min and twice in xylene for 10 min, and finally three times (45, 60, and 60 min, respectively) in paraffin at 65°C for waxing. After dehydration and wax immersion, the brain tissues of the mice were quickly put into an embedded frame containing 10 ml wax solution at 65°C. The wax solution was cooled to −20°C, and the brain tissues of the mice were solidified. The brain tissues in wax were cut into slices of 4 μm thickness using a paraffin-sectioning machine.

### 2.5 Immunohistochemical Staining

The slice on the slide was soaked in xylene I, xylene II, xylene III, and anhydrous ethanol for 10 min successively, then soaked in 95% ethanol and 90% ethanol three times, and finally washed with PBS solution three times (3 min for each time). Then, the slide was placed in citric acid solution and maintained at 105°C for 10 min in a pressure cooker for antigen repairing. At last, the slide was placed in PBS solution (pH = 7.4) and washed three times (5 min for each time) on an automatic shaker. Endogenous peroxidase blocker (3% H_2_O_2_ deionized water) was carefully added to the tissue on the slide for 10 min, and the slide was then placed in PBS solution (pH = 7.4) and washed with PBS three times again.

After the tissue on the slide was sealed with the sealing solution for 15 min at room temperature, the first antibody of rabbit anti-beta-amyloid 1–40 (CT) antibody (dilution of 1:500) was added on the tissue on the slide and placed in a dark box overnight at 4°C. Then, a secondary antibody corresponding to the first antibody, biotinylated antibody labeled with HRP, was dropped onto the tissue and incubated for 30 min at room temperature. Then, the slides were placed in PBS solution and washed on a shaker three times (5 min for each time). After the slides were slightly dried, the newly prepared DAB staining solution was carefully added to cover the whole tissue. When the tan-positive reaction was observed under a microscope, the slides were rinsed with running water to stop the staining.

After the slide was dehydrated with 90% ethanol, 95% ethanol, 100% ethanol, n-butanol, xylene I, and xylene II, it was sealed with neutral gum and observed using an inverted fluorescence microscope.

### 2.6 Hematoxylin-Eosin Staining

After the dewaxing and hydrating of the slide similar to immunohistochemical staining, the slide was put into hematoxylin-dyeing solution and stained for 3–5 min. The clean slide was soaked in 85% ethanol for 5 min successively, and then it was put into eosin staining solution for another 5 min and washed with double steaming water. Then, the slide was dehydrated and sealed and observed using an inverted microscope.

### 2.7 Silver Staining for Neurofibrillary Tangles

After the dewaxing and hydrating of the slide similar to immunohistochemical staining, the slide was put into silver glycine dye C to stain for 5 min, and then it was rinsed with double steaming water three times. Then, the slide was soaked in silver glycine solution B for 3–5 min. At last, the slide was put into preheated silver glycine dye solution A I (45°C) and preheated silver glycine dye solution A II (45°C) for another few seconds; then, the slide was washed with distilled water. Finally, the slide was dehydrated and sealed and observed using an inverted microscope.

### 2.8 Western Blot

The Western blot analysis was achieved as the reported method with minor modifications (Meng et al., 2017). Briefly, the mice were sacrificed by neck removal; the hippocampus and cortex tissues were taken out and washed with cold PBS 3 times and put into a glass homogenizer. After 0.25–0.3 ml of tissue lysate was added, the tissues of about 20 mg were thoroughly ground to form a homogenate. The lysed tissue homogenate was put into the centrifuge and centrifuged at 12000 RPM and 4°C for 15 min. The supernatant part of the centrifuge tube was collected as a total protein solution. After the proteins were separated on SDS-PAGE gels and transferred to polyvinylidene fluoride membranes, the membranes were incubated with first antibodies of rabbit calpain two polyclonal antibody (dilution of 1:1500), rabbit SELK polyclonal antibody (dilution of 1:1500), and rabbit anti-β-actin (dilution of 1:5000) respectively, followed by the incubation of secondary antibodies of HRP-conjugated goat anti-rabbit antibody (dilution of 1:200). The membranes were treated with the ECL reagents (Tanon, Shanghai, China) and visualized using a Tanon 5200 Multi Chemiluminescent System (Tanon).

### 2.9 Statistical Analysis

SPSS 19.0 software was used, and means between two treatments were compared by the Student’s *t*-test, or means among three or more treatments were compared by one-way ANOVA followed by the least significant difference test. Data were represented as the means ± SEM.

## 3 Results

### 3.1 The Effect of BBMH Administration on the Learning and Memory Abilities of Three Kinds of AD Mice

#### 3.1.1 The Escape Latency of Mice

**FIGURE 1:**
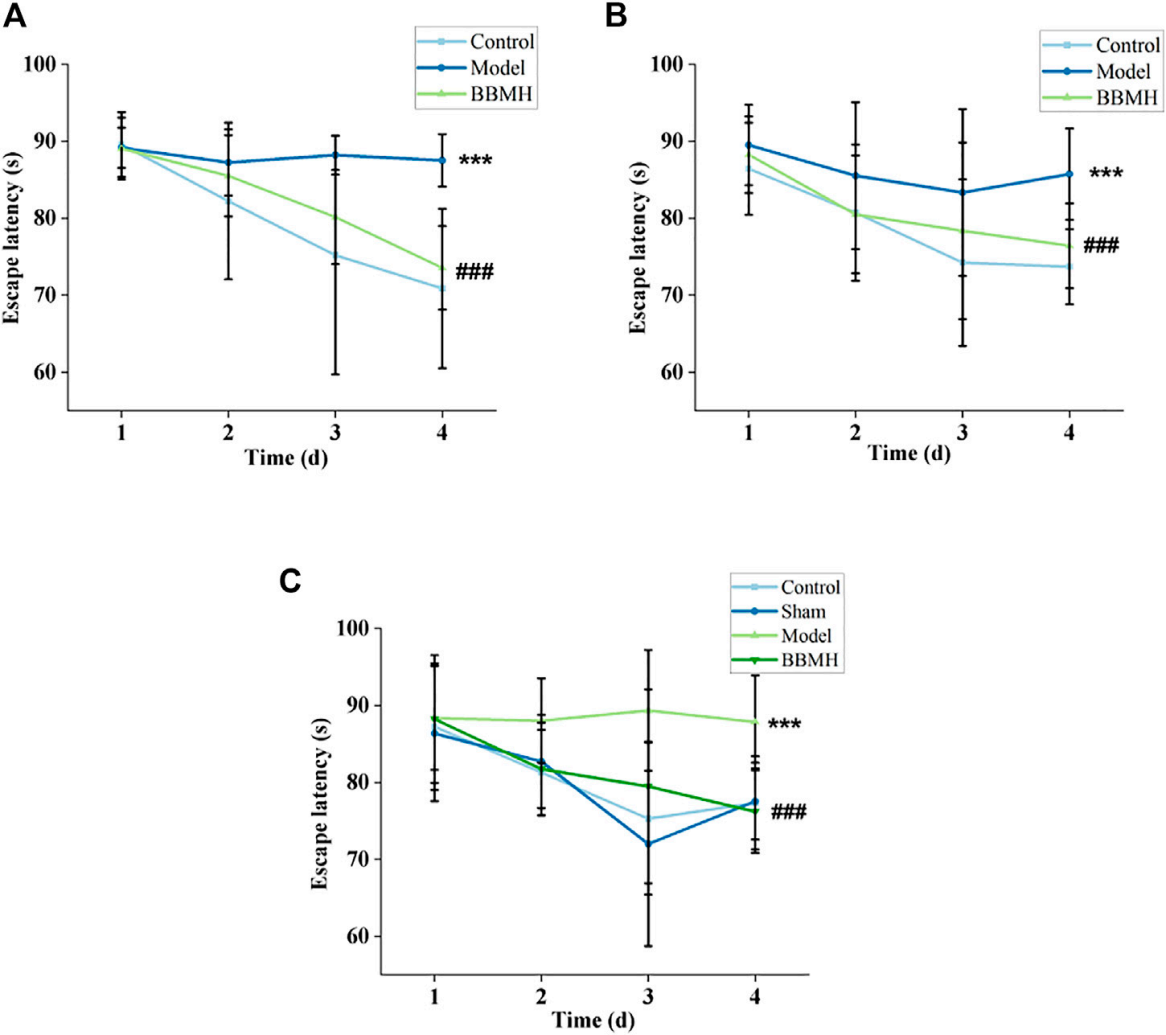
Effects of BBMH administration on the escape latency of three kinds of AD model mice. The line charts show the relationship between the escape latency and the days of training of the mice. **(A)** Control, APP/PS1, and BBMH mice, respectively. **(B)** Control, Alu-Gal, and BBMH mice, respectively. **(C)** Control (and/or sham operation mice), Alu-AD, and BBMH mice, respectively. Data were represented as the means; ***p < 0.001, **p < 0.01, *p < 0.05 vs. control and AD mice; ^###^ *p* < 0.001, ^##^ *p* < 0.01, ^#^ *p* < 0.05 vs. AD mice and BBMH-administered mice.

The escape latency of the APP/PS1 model group was not changed significantly after every day’s training and learning; on the other hand, that of their control mice group (normal mice) was significantly shortened, indicating that their positioning learning and memory abilities decreased significantly (Figure 1A). However, the escape latency of the BBMH-treated AD mice group was gradually shortened and approached to control mice after 3 days of training, indicating that the positioning learning and memory abilities of AD mice were greatly improved after BBMH administration.

It also could be found that the escape latency of the Alu-Gal AD mice group (Figure 1B) and Alu AD mice group (Figure 1C) did not change significantly after 3 days of training and learning, indicating that the learning and memory abilities of them also decreased significantly. However, the escape latency of the BBMH-treated Alu-Gal mice group (Figure 1B) and Alu mice group (Figure 1C) was gradually shortened and approached to the control mice group (and/or sham operation group) on the 4th day, indicating that the learning and memory abilities of BBMH-treated Alu-Gal AD mice and Alu AD mice were greatly improved after BBMH administration.

#### 3.1.2 The Time in the Target Quadrant and the Number of Crossing Platform of Mice

**FIGURE 2:**
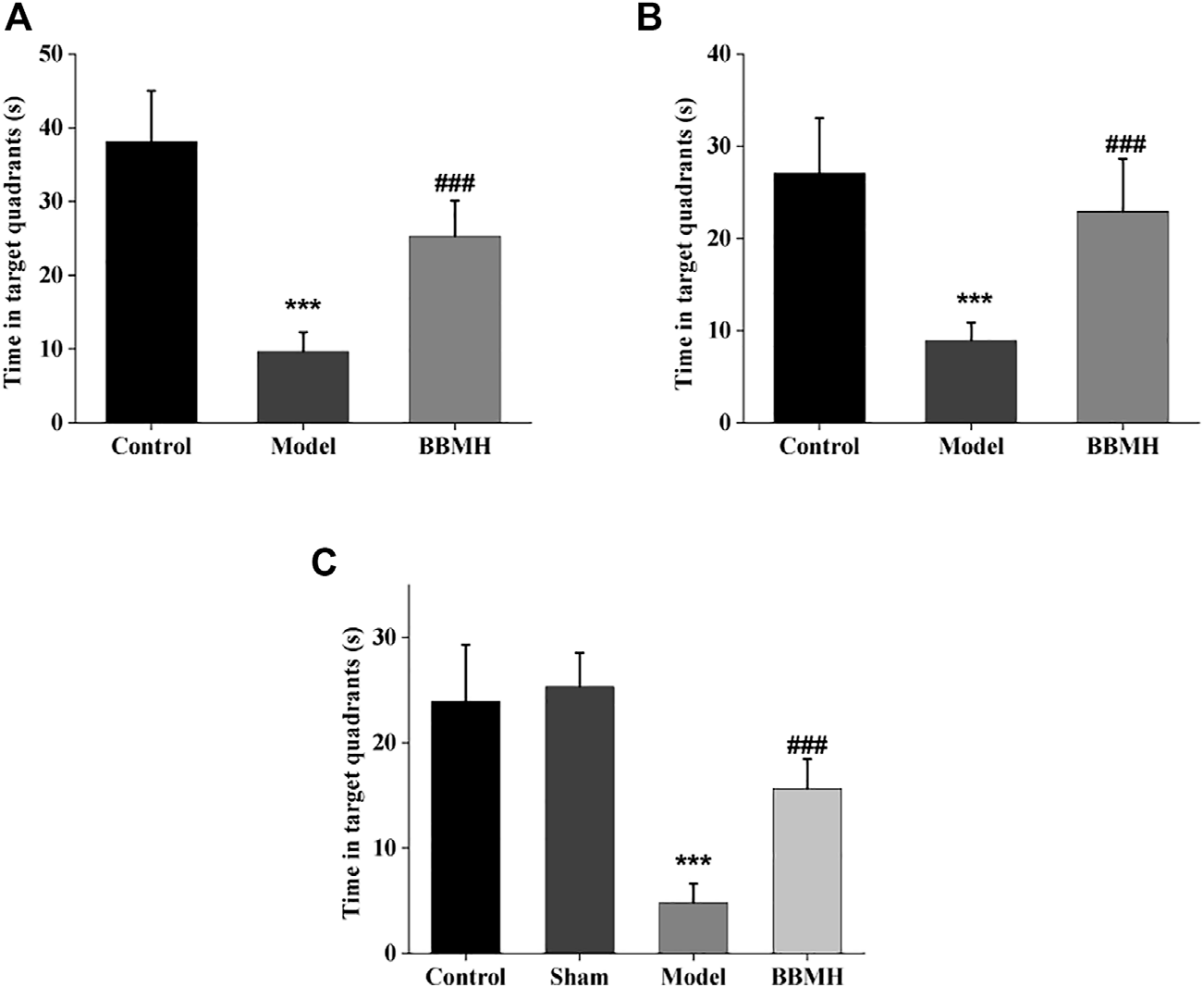
Effects of BBMH administration on the time in the target quadrant of three kinds of AD model mice. The histograms show the time in the target quadrant of mice after 4 days of training, respectively. **(A)** Control, APP/PS1, and BBMH mice. **(B)** Control, Alu-Gal, and BBMH mice. **(C)** Control (and/or sham operation mice), Alu, and BBMH mice. Data were represented as the means ± SEM; ***p < 0.001, control group vs. AD group; ^###^ *p* < 0.001, AD group vs. BBMH group.

**FIGURE 3:**
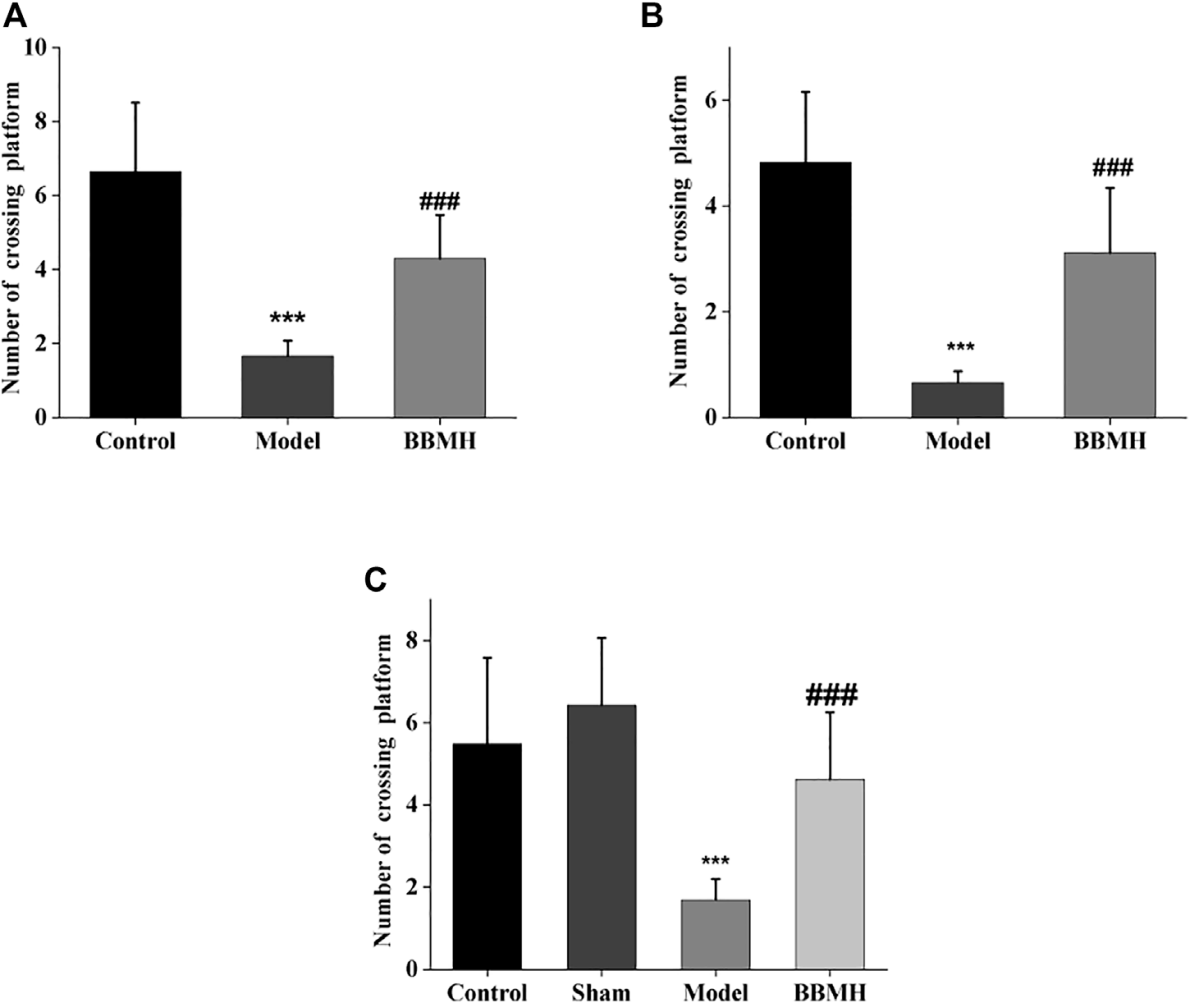
Effects of BBMH administration on the number of crossing platform of three kinds of AD model mice. The histograms show the number of crossing platform of mice after 4 days training, respectively. **(A)** Control, APP/PS1, and BBMH mice. **(B)** Control, Alu-Gal, and BBMH mice. **(C)** Control (and/or sham operation mice), Alu AD, and BBMH mice. Data were represented as the means ± SEM; ***p < 0.001, control group vs. AD group; ^###^ *p* < 0.001, AD group vs. BBMH group.

After 4 days of learning and training, the time in the target quadrant and the number of crossing platform of APP/PS1 mice, Alu-Gal AD mice, and Alu AD mice were significantly lower than those of their control mice groups on the 5th day (Figure 2 and Figure 3), indicating that the spatial learning and memory abilities of the three kinds of AD model mice were significantly decreased. After BBMH administration, the time in the target quadrant and the number of crossing the platform of the three kinds of BBMH-treated AD mice groups were significantly increased (Figure 2 and Figure 3), indicating that BBMH could significantly improve the learning and memory abilities of the three kinds of AD mice.

### 3.2 The Effects of BBMH on the Formation of Aβ Deposition in the Brain

**FIGURE 4:**
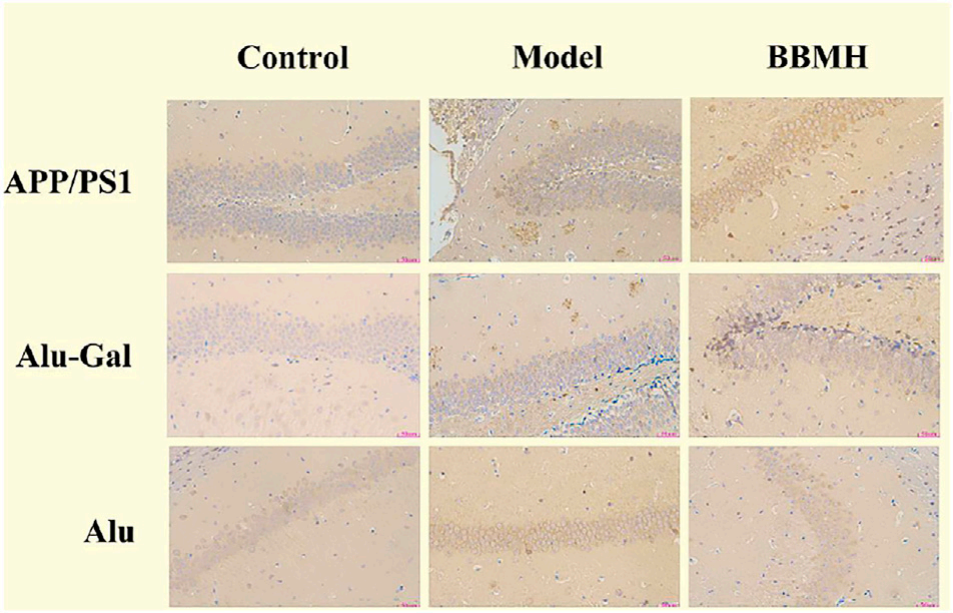
Effects of BBMH administration on Aβ plaque formation in the hippocampal tissue of three kinds of AD model mice. Aβ plaque formation was shown by immunohistochemical staining. (APP/PS1) Control, APP/PS1, and BBMH mice. (Alu-Gal) Control, Alu-Gal, and BBMH mice. (Alu) Control (sham operation mice), Alu AD, and BBMH mice.

Aβ plaque appeared in the hippocampal tissues of the APP/PS1 model mice group and the Alu-Gal AD model group (Figure 4). On the other hand, there was no Aβ plaque in the hippocampal tissues of mice in the control groups. Only few Aβ plaques could be observed in the brain hippocampal tissues of the BBMH group, suggesting that BBMH can inhibit the production of Aβ deposition in the brain hippocampal tissues of APP/PS1 mice. No Aβ plaque was discovered in Alu AD model mice, suggesting that the stereotactic injection of aluminum chloride into the brain can only produce AD-alike model mice since no Aβ deposition appeared.

### 3.3 The Effects of BBMH Administration on the Hippocampal Pyramidal Cells

**FIGURE 5:**
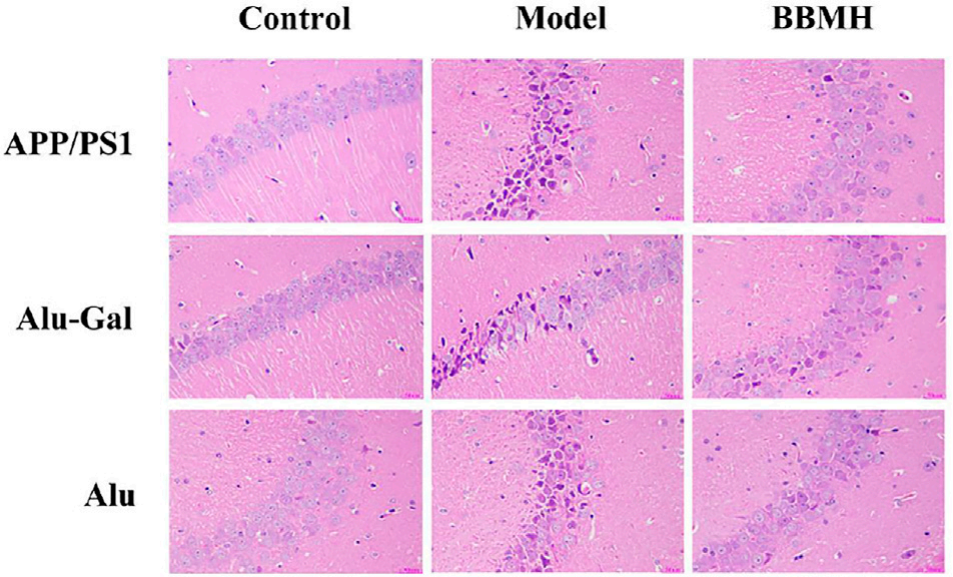
Effects of BBMH administration on the states of hippocampal pyramidal cells in three kinds of AD model mice. The changes in pyramidal cells were shown by hematoxylin-eosin staining. (APP/PS1) Control, APP/PS1, and BBMH mice. (Alu-Gal) Control, Alu-Gal, and BBMH mice. (Alu) Control (sham operation mice), Alu AD, and BBMH mice.

The pyramidal cells (neurons) in the hippocampal tissues of mice in the control mice groups (normal mice for APP/PS1 mice and Alu-Gal AD mice and sham operation mice for Alu AD mice) of three kinds of AD model mice were in normal shape, orderly arranged, and uniformly colored, without pathological features (Figure 5). The pyramidal cells in the hippocampal tissues of the three kinds of AD model mice groups showed more lesions, irregular cell boundaries, large intercellular space, and dark red staining after lysis. In all of the three kinds of BBMH-treated AD mice groups, only a few pyramidal cells showed irregular shape, indicating that BBMH can alleviate the hippocampal neuron damage of hippocampal tissues in AD mice.

### 3.4 The Effects of BBMH on the Formation of Neurofibrillary Tangles

**FIGURE 6:**
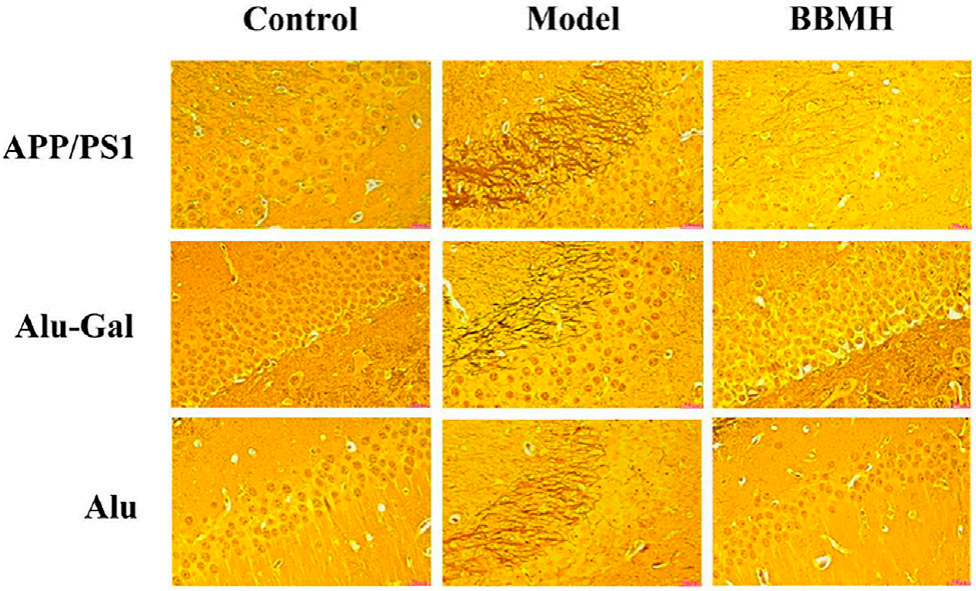
Effects of BBMH administration on the formation of NFTs in three kinds of AD model mice. NFTs were shown by silver staining. (APP/PS1) Control, APP/PS1, and BBMH mice. (Alu-Gal) Control, Alu-Gal, and BBMH mice. (Alu) Control (sham operation mice), Alu AD, and BBMH mice.

As shown in Figure 6, no neurofibrillary tangles (NFTs) appeared in the hippocampal tissues of the control mice groups in the three kinds of AD model mice (Figure 6). On the other hand, many brown neurofibrillary tangles appeared in the hippocampal tissues of three kinds of AD model mice groups. Among them, most brown neurofibrillary tangles appeared in the hippocampal tissues of APP/PS1 model mice, followed by Alu-Gal AD model mice, and least for Alu AD model mice, indicating that APP/PS1 model mice had the highest degree of neuropathy. After BBMH was administered to three kinds of AD mice, the neurofibrillary tangles in the hippocampal tissues of three kinds of BBMH-treated mice groups were significantly reduced. The neurofibrillary tangles completely disappeared in BBMH-treated Alu AD mice and Alu-Gal AD mice, and only a small number of neurofibrillary tangles could be found in BBMH-treated APP/PS1 mice. These results indicate that the neurofibrillary tangles in the hippocampal tissues of AD mice can be reduced by BBMH administration.

### 3.5 The Effects of BBMH on the Expression of Proteolytic Enzyme, Calpain

**FIGURE 7:**
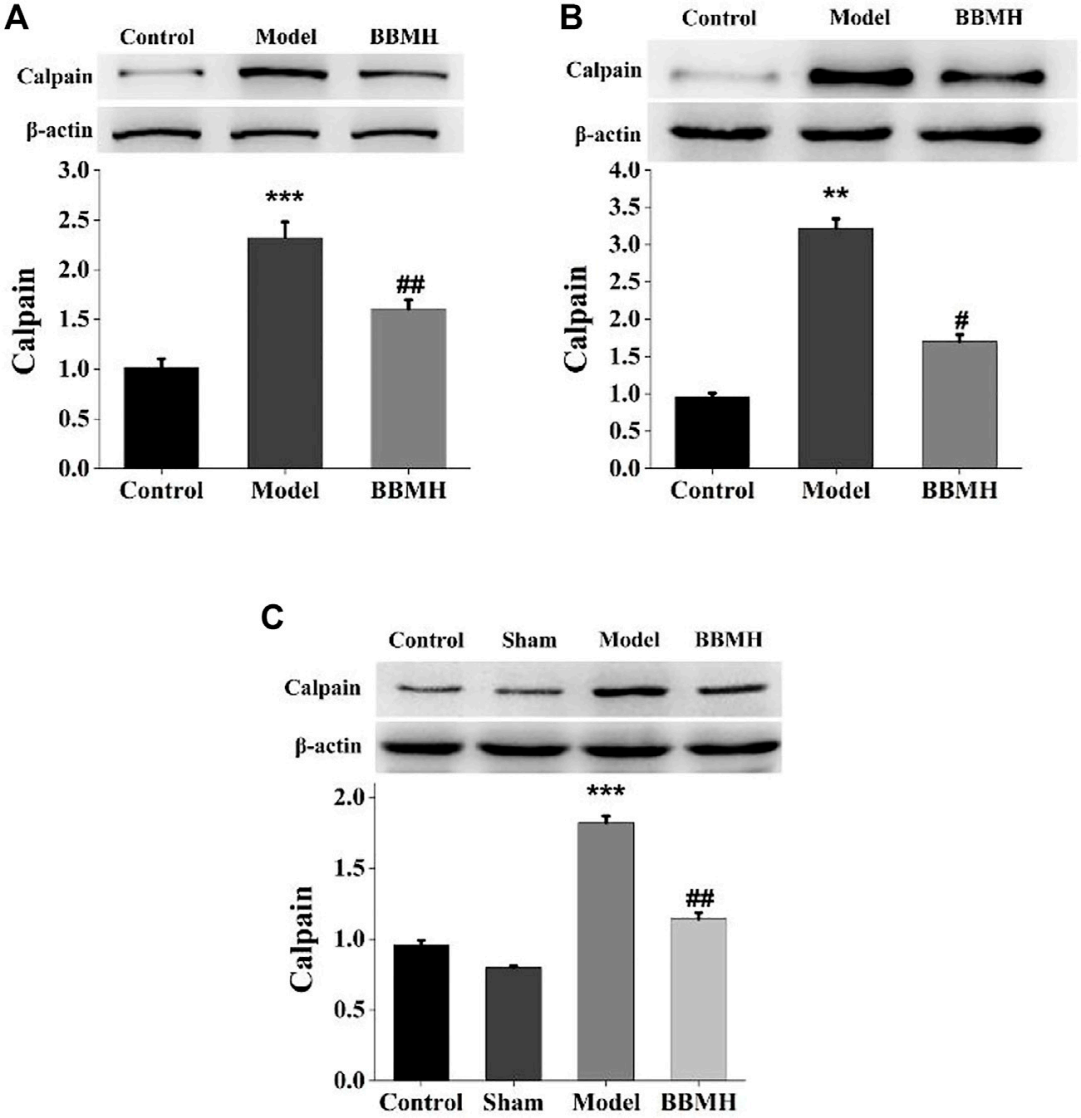
Effects of BBMH administration on the expression level of calpain in the brain tissue of three kinds of AD model mice. **(A)** Expression of calpain and β-actin in control, APP/PS1, and BBMH mice, respectively. **(B)** Expression of calpain and β-actin in control, Alu-Gal mice, and BBMH mice, respectively. **(C)** Expression of calpain and β-actin in control, Alu, and BBMH mice, respectively. The monomeric form of calpain protein and the β-actin band were quantitatively measured by ImageJ software. Data of the relative amount of calpain to β-actin were represented as the means ± SEM; ***p < 0.001, **p < 0.01, vs. control [sham operation mice in **(C)**] and AD mice; ^##^ *p* < 0.01, ^#^ *p* < 0.05, vs. AD mice and BBMH-administered mice.

Since BBMH belongs to bisbenzylisoquinoline alkaloids, which are Ca^2+^ channel antagonists (Meng et al., 2019), the expression level of Ca^2+^-dependent proteolytic enzyme, calpain, in the brain tissues of the mice was investigated by Western blot. It was shown that the expression level of calpain in three kinds of AD model mice was significantly increased compared to that of their control mice and that in three kinds of BBMH-treated AD mice was significantly decreased (Figure 7).

### 3.6 The Effects of BBMH on the Expression of Selenoprotein SelK

**FIGURE 8:**
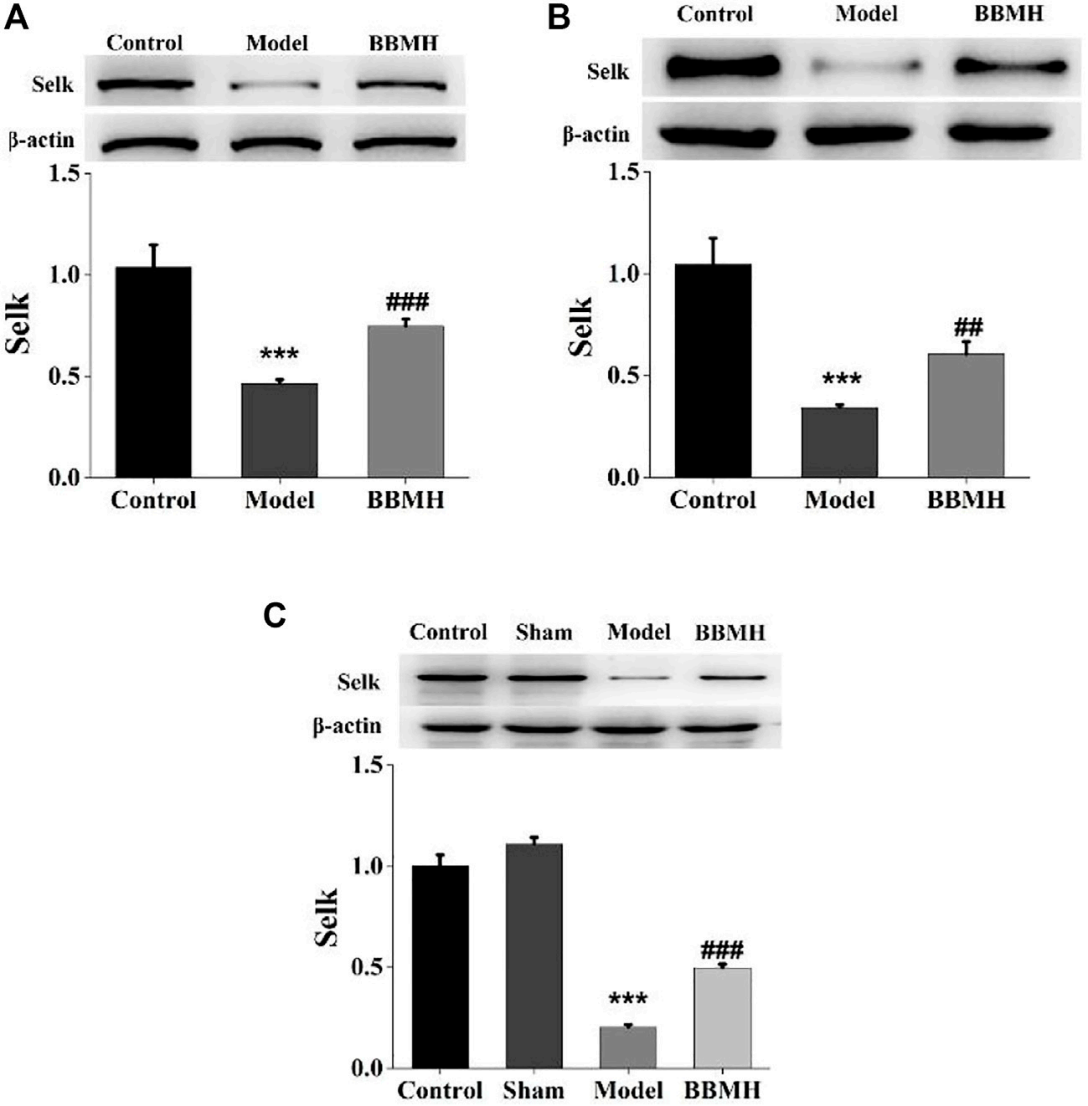
Effects of BBMH administration on the expression level of selenoprotein SelK in the brain tissue of three kinds of AD model mice. **(A)** Expression of selenoprotein SelK and β-actin in control, APP/PS1, and BBMH mice, respectively. **(B)** Expression of selenoprotein SelK and β-actin in control, Alu-Gal, and BBMH mice, respectively. **(C)** Expression of selenoprotein SelK and β-actin in control, Alu, and BBMH mice, respectively.

The expression of selenoprotein SelK, one of the proteolytic target proteins of calpain (Huang et al., 2011), in the brain tissues of the mice was also tested by Western blot. It was shown that the expression level of selenoprotein SelK in three kinds of AD model mice was significantly decreased compared to that of their control mice and that of SelK was significantly increased in three kinds of BBMH-treated AD mice (Figure 8).

## 4 Discussion

Bisbenzylisoquinoline (BBI) alkaloids belong to the very large isoquinoline alkaloid family, and everyone consisted of two benzylisoquinoline parts connected by oxygen bridges. Most BBI, including fangchinoline, cyclanoline, dauricine, liensinine, isoliensinine, and neferine, exhibited good inhibitory activities of acetylcholinesterase (AChE) and butyrylcholinesterase (BChE) and also demonstrated noncompetitive enzyme inhibition (Lin et al., 2013; Jung et al., 2015; Li et al., 2019; Mir et al., 2021).

Microglial cells play an important role in brain tissues through mediating neuroinflammation in Alzheimer’s disease (AD) by the production of a series of proinflammatory mediators as well as clearance of Aβ peptides and senile plaques. It has been found that tetrandrine decreases the expression of proinflammatory mediators such as IL-1β and TNF-α in microglial cells by inhibition of NF-κB activation (He et al., 2011). Dauricine can significantly improve cognitive impairments in 3xTg-AD mice by decreasing Aβ plaques and hyperphosphorylated Tau and increasing the hippocampal ATP level by modulating the tricarboxylic acid cycle, synaptic vesicle cycle, glycolysis, and gluconeogenesis in 3xTg-AD mice (Chen Y. et al, 2021). It is proposed that most of the BBI compounds play physiological roles partly by the inhibition of the increasing of the Ca^2+^ level in the cells and the subsequent phosphorylation of CaMKII as calmodulin-binding agents (Meng et al., 2019).

Thus, BBI is a multi-target anti-Alzheimer’s disease compound based on the aforementioned analysis. In this study, BBMH exhibited significant anti-Alzheimer’s disease functions possibly through its multi-target actions. BBMH can also inhibit the expression of Ca^2+^-dependent proteolytic enzyme, calpain, by regulating the intracellular calcium homeostasis, and increase the expression of SelK, a special selenoprotein expressed mainly in immune cells, thereby enhancing the clearance abilities of Aβ and Tau protein in brain tissues through increasing the phagocytosis of microglial cells and macrophages migrated to the brain.

## Data Availability

The original contributions presented in the study are included in the article/Supplementary Materials; further inquiries can be directed to the corresponding author.
